# Nutzung von Gesundheitsinformationen im Internet: personenbezogene und motivationale Einflussfaktoren

**DOI:** 10.1007/s00103-020-03144-5

**Published:** 2020-05-04

**Authors:** Elena Link, Eva Baumann

**Affiliations:** Institut für Journalistik und Kommunikationsforschung, Hochschule für Musik, Theater und Medien Hannover, Expo Plaza 12, 30539 Hannover, Deutschland

**Keywords:** Gesundheitsbezogene Online Recherche, Gesundheitsförderung, Befähigte Patienten, Unsicherheitsbewältigung, Health-realted online search, Health promotion, Empowered patients, Uncertainty management

## Abstract

**Hintergrund und Ziele:**

Die Übernahme einer aktiven Patient*innen-Rolle kann nur auf einer entsprechenden Informationsgrundlage gelingen. Die eigene Suche nach Gesundheitsinformationen im Internet kann zu einer solchen adäquaten Wissensbasis beitragen. Daher erscheint es zentral zu fragen, was die derzeitige Nutzung von Onlineangeboten und Gesundheits-Apps in Deutschland auszeichnet und welche personenbezogenen und motivationalen Einflussfaktoren beeinflussen, ob und wie häufig die Bürger*innen online nach Gesundheitsinformationen suchen oder Gesundheits-Apps nutzen.

**Methoden:**

Zur Beantwortung der Fragestellungen wurde eine an der deutschen Gesamtbevölkerung stratifizierte Onlinebefragung (*N* = 3000) durchgeführt. Der Fragebogen erfasste die gesundheitsbezogene Nutzung des Internets und von Apps sowie mögliche personenbezogene und motivationale Einflussfaktoren. Die Bedeutung dieser Faktoren für die Internetnutzung wurde mittels Regressionsanalysen ermittelt.

**Ergebnisse:**

Die gesundheitsbezogene Informationssuche mittels Internet ist in Deutschland weitverbreitet, während Gesundheits-Apps noch deutlich seltener genutzt werden. Am häufigsten werden auf Gesundheitsportalen Informationen zu Krankheitssymptomen gesucht. Die Zuwendung zum Internet wird besonders durch motivationale Faktoren geprägt. Akute Betroffenheit von körperlichen Beschwerden und entsprechende Informationskompetenzen sind die einflussreichsten Faktoren für die Internetnutzung.

**Diskussion:**

Um die mit der Informationssuche im Internet verbundenen Potenziale zu entfalten, ist es notwendig, dass sich Bürger*innen zur eigenen Suche motiviert und befähigt fühlen. Besonders entsprechende Fähigkeiten gilt es zu fördern, um das Risiko der Verstärkung informationaler, gesundheitlicher und sozialer Unterschiede zu reduzieren.

## Abstract (en)

**Background and Objectives:**

Taking an active role in healthcare requires comprehensive health knowledge. One’s own online search for health information can contribute to an appropriate information base. Therefore, it seems essential to determine the extent to which online services and health apps are currently used in Germany as well as to analyze which personal and motivational factors influence whether and how often the German public search for health information online or use health apps.

**Methods:**

We conducted an online survey among the German public in a sample stratified by demographic characteristics (*N* = 3000). The questionnaire measured the health-related use of the Internet and health apps as well as possible personal and motivational factors. The relevance of these factors for explaining the Internet use for health-related purposes was determined by regression analyses.

**Results:**

While health-related information searches using the Internet are widespread in Germany, health apps are used less frequently. In particular health information platforms are most frequently used to search for information on disease symptoms. Turning to the Internet for health-related purposes is mainly influenced by motivational factors. Having current health problems and possessing information-seeking skills are particularly influential factors for an individual’s Internet use.

**Conclusions:**

In order to unfold the potential associated with health-related online information-seeking behaviors, it is necessary that people are motivated and feel competent to gather online information themselves. In particular, the corresponding skills need to be promoted in order to reduce the risk of increasing informational, health, and social disparities.

## Hintergrund

Für die Bewältigung einer Erkrankung und den Erhalt der Gesundheit sind Zugang und angemessene Nutzung von gesundheitsbezogenen Informationen von hoher Bedeutung. So gewonnenes Wissen dient als Grundlage für gesundheitsrelevante Entscheidungen und hilft bei der Bewältigung emotionaler Belastungen und subjektiv wahrgenommener Unsicherheit [1]. Die Bereitschaft und Fähigkeit zur aktiven Auseinandersetzung mit Gesundheitsinformationen der Rezipient*innen einerseits und die Bereitstellung qualitätsgesicherter Informationen andererseits sind Voraussetzung für die Prävention sowie die Befähigung jedes oder jeder Einzelnen, sich aktiv in die eigene Gesundheitsversorgung einzubringen – was vor allem aufgrund der geforderten Verantwortungsübernahme mündiger Patient*innen zunehmend bedeutsam erscheint [2–4]. Demnach ist das Informationshandeln als kommunikative und kognitive Aktivität des Suchens, Bewertens und Interpretierens von gesundheitsbezogenen Informationen [5] zentral für die Förderung und den Erhalt der Gesundheit.

Vor diesem Hintergrund sind ein grundlegendes Verständnis und fundiertes Wissen darüber, wer aufgrund welcher Motive nach Gesundheitsinformationen sucht und welche Kanäle dafür relevant sind, elementar [6, 7]. Dem Internet kommt in diesem Zusammenhang eine besondere und perspektivisch weiter steigende Bedeutung zu (im Überblick vgl. [8]). Der vorliegende Artikel fokussiert die Bedeutung digitaler Angebote zur Information und Unterstützung. Dazu zählen Onlineangebote, die zur Suche nach oder zum Austausch über Gesundheitsinformationen herangezogen werden [9], ebenso wie Gesundheits-Apps, denen im Zuge der Omnipräsenz des Smartphones [10] eine zunehmende Bedeutung für das Monitoring und die Förderung der eigenen Gesundheit sowie das Krankheitsmanagement zukommt (z. B. [11]). Diesen Angeboten wird das Potenzial zugeschrieben, Patient*innen bei gesundheitsbezogenen Entscheidungen zu unterstützen und sie mit Informationen zur Vor- und Nachbereitung eines Arzttermins zu versorgen [12]. Zudem sollen solche Tools und Angebote dazu beitragen, dem Individuum mehr Kontrolle über die eigene Gesundheitsversorgung und mehr Wissen über den eigenen Gesundheitszustand zu geben [13]. Dies kann auch damit einhergehen, Voraussetzungen für die Übernahme einer aktiven Rolle in der eigenen Gesundheitsversorgung zu schaffen und soziale Ungleichheiten hinsichtlich des Zugangs zu Gesundheitsinformationen zu reduzieren [13–15]. Speziell Gesundheits-Apps bieten neben den Potenzialen für die Selbstbestimmung und -verantwortung (Empowerment) des oder der Einzelnen auch die Möglichkeit, eigenes Verhalten (z. B. Sport, Ernährungsweisen oder Gesundheitsdaten) zu dokumentieren, zu überwachen und mit anderen zu teilen [16]. Davon kann ebenfalls eine Unterstützungsfunktion für eine gesunde Lebensweise und den Umgang mit Erkrankungen ausgehen.

Den Potenzialen durch das umfangreiche, vielfältige und aktuelle Angebot an Gesundheitsinformationen und die Möglichkeit der aktiven und zielgerichteten Onlinerecherche und konstruktiven Nutzung von Apps steht die Herausforderung gegenüber, hochwertige Informationen bzw. Apps zu finden, vertrauenswürdige Quellen und Anbieter zu identifizieren, mit gegebenenfalls widersprüchlichen Informationen umzugehen und komplexe Fachinformationen zu verstehen. Denn längst nicht alle Personen sind fähig und gewillt, im Internet nach gesundheitsbezogenen Informationen zu suchen, sie kritisch zu bewerten und Gesundheits-Apps beispielsweise zum Monitoring des eigenen Verhaltens zu nutzen. Es stellt sich daher die Frage, wer durch die Angebote erreicht wird, damit zu den sogenannten Gesundheits-Onlinern zählt und welche Faktoren die Zuwendung und Nutzung beeinflussen. Erklärungsansätze für das gesundheitsbezogene Informationshandeln bieten erste Hinweise auf relevante Prädiktoren [3, 17–19]. Bislang gibt es jedoch nur relativ wenige Modelle, die das gesundheitsbezogene Informationshandeln erklären (in der Übersicht [3]), und kein einheitliches Verständnis der relevanten Prädiktoren. Vor allem auf die Besonderheiten des Internets beziehen sich bisher wenige Ansätze [14, 20].

Zu den bislang als relevant identifizierten Einflussfaktoren der gesundheitsbezogenen Informationssuche zählen *personenbezogene Merkmale* wie das Alter, Geschlecht und der Bildungsgrad [9, 21–23]. Zudem gilt der *Gesundheitszustand* als ein situativer Auslöser von Informationsbedürfnissen [24, 25] und erste Studien deuten darauf hin, dass das Internet vor allem eine Quelle für besonders stark an Gesundheitsthemen interessierte Personen (*Hochinvolvierte*) und *chronisch Erkrankte* darstellt [21, 26]. Ebenso sind *motivationale Faktoren* relevant. Zu diesen zählen wahrgenommene internetbezogene *Informationskompetenzen *– also die Frage, inwiefern eine Person sich in der Lage sieht, im Internet relevante Informationen zu finden, zu verstehen und zu bewerten. Die Suche nach gesundheitsbezogenen Informationen im Internet gilt als besonders voraussetzungsreich, da die Fülle an Informationen und die schwankende Qualität das Auffinden hochwertiger und vertrauenswürdiger Quellen erschweren [27, 28]. Als ebenso vielfältig und in der Qualitätsbewertung herausfordernd ist auch der Markt an Gesundheits-Apps zu bewerten [16].

Ein weiterer motivationaler Faktor ist das *gesundheitsbezogene Informationsinteresse* als Bestandteil des generellen Gesundheitsbewusstseins [29]. Es beschreibt, wie hoch der Wunsch nach Gesundheitsinformationen und daraus resultierendem Wissen über die eigene Gesundheit ist. Zudem beeinflusst es, wie stark Informationsbedürfnisse ausgeprägt sind und wie viel in die Informationssuche investiert wird [20]. Ein ausgeprägtes Interesse macht die Auseinandersetzung mit gesundheitsbezogenen Informationen generell wahrscheinlicher, beeinflusst die Anzahl der genutzten Informationskanäle sowie die Vielfalt der gesuchten Themen [29–33]. Als motivationalen Faktor berücksichtigen manche Modelle zudem die *Einstellung gegenüber bestimmten Informationsquellen* [17, 18, 34]. Dabei wird u. a. auf den Einfluss der Einstellung von Rezipient*innen verwiesen, inwiefern sie eine spezifische Quelle als nützlich wahrnehmen, um ihre Informationsbedürfnisse zu befriedigen [17]. Einen zentralen Aspekt der Nützlichkeit stellt vor dem Hintergrund der Qualitätsdebatte um Gesundheitsinformationen das Vertrauen in das Internet dar, da dieses beschreibt, inwiefern mediale Gesundheitsinformationen als zutreffend angesehen und relevant für die eigene Situation bewertet werden [20].

Auf Basis der dargestellten Prädiktoren verfolgt der Beitrag das Ziel, zu einem besseren Verständnis der Nutzung digitaler Gesundheitsangebote in Form der Informationssuche im Internet und der Nutzung von Gesundheits-Apps beizutragen. Dazu wird zunächst die gesundheitsbezogene Internetnutzung beschrieben und anschließend identifiziert, welche personenbezogenen und motivationalen Faktoren die Internetnutzung sowie die Nutzung von Gesundheits-Apps beeinflussen. Hieraus resultieren folgende Forschungsfragen:

1. Was charakterisiert die Nutzung digitaler Gesundheitsangebote?
2. Welche personenbezogenen und motivationalen Einflussfaktoren beeinflussen,
  - ob und wie häufig die Bürger*innen im Internet nach Gesundheitsinformationen suchen und
  - ob sie Gesundheits-Apps nutzen?

## Methode

Zur Beantwortung der Forschungsfragen wurde im Dezember 2018 eine Onlinebefragung durchgeführt (*N* = 3000). Mittels eines Online.Access-Panel wurde eine für die Internetnutzer*innen in der deutschen Bevölkerung nach Alter (18 bis 69 Jahre), Geschlecht, Bildung und Region stratifizierte Stichprobe rekrutiert. Die zugehörige Grundgesamtheit der Befragung stellt somit der Anteil der Internetnutzer*innen in Deutschland dar. Dieser liegt mittlerweile bei 90,3 %. Ein etwas höherer Anteil der Internetnutzer*innen sind Männer und Jüngere (vollständige Abdeckung der 18- bis 29-Jährigen, während nur 64,8 % der über 70-Jährigen das Internet nutzen, [10]). Die in der vorliegenden Studie Befragten waren durchschnittlich 44,3 Jahre alt (*SD* = 14,28) und die Hälfte der Proband*innen war weiblich. Die verschiedenen Bildungsabschlüsse waren relativ gleichverteilt (30,4 % Hauptschulabschluss; 33,3 % mittlere Reife; 36,3 % mindestens (Fach‑)Abitur).

**Figure 1:**
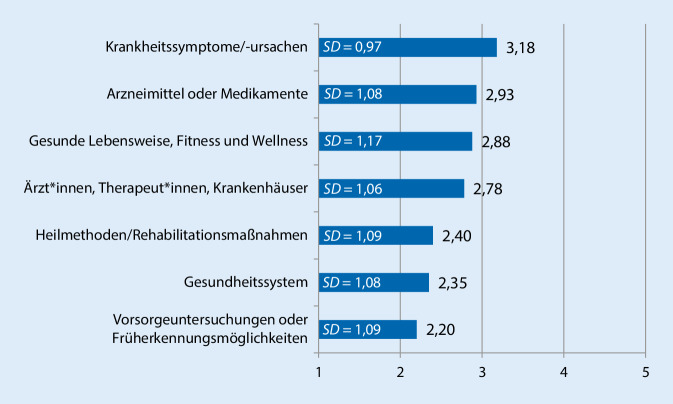

**Figure 2:**
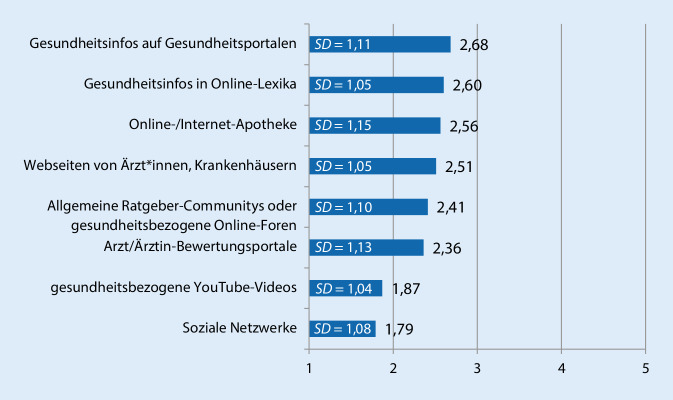

Das Erhebungsinstrument erfasste verschiedene Aspekte der gesundheitsbezogenen Internetnutzung sowie mögliche personenbezogene und motivationale Einflussfaktoren. Zur Beschreibung der *gesundheitsbezogenen Internetnutzung *wurde erfasst, ob die Proband*innen innerhalb des letzten Jahres im Internet nach Gesundheitsinformationen gesucht hatten und an wie vielen Tagen innerhalb des letzten Monats dies der Fall war [35]. Zudem wurde für die „Gesundheits-Onliner“ erfasst, wie häufig sie sich über unterschiedliche Themen informierten (Abb. 1) und welche spezifischen Angebote sie dafür nutzten (Abb. 2). Die ausgewählten Themen sowie Onlineangebote orientieren sich an der Abfrage der Nutzung unterschiedlicher Informationsangebote in der 22. Welle des Gesundheitsmonitors der Bertelsmann Stiftung [30]. Die Häufigkeit der Suche nach bestimmten Themen und die Nutzung der einzelnen Angebote wurde jeweils auf einer 5er-Skala von „nie“ (1) bis „sehr häufig“ (5) erfasst. Zusätzlich wurde mittels einer weiteren Frage erhoben, ob die Befragten Gesundheits-Apps verwenden.

Als *personenbezogene Einflussfaktoren* wurden das Alter, Geschlecht und der Bildungsstand der Befragten erhoben. Zudem wurde erfragt, ob sich die Proband*innen derzeit wegen körperlicher oder psychischer Beschwerden in ärztlicher Behandlung befinden. Um zusätzlich den subjektiven Gesundheitszustand zu erfassen, wurden die Befragten in Anlehnung an die Operationalisierung des Gesundheitsmonitors sowie des in den USA etablierten HINTS-Fragebogens^1^ gebeten, ihren allgemeinen Gesundheitszustand auf einer 5er-Skala von „schlecht“ bis „ausgezeichnet“ zu bewerten.

Als *motivationale Einflussfaktoren *wurde die Kompetenzeinschätzung zur Onlinesuche nach Gesundheitsinformationen mittels E‑Health Literacy Scale (E-Heals) [27, 36] erfasst. Diese Skala umfasst 8 Items (z. B.: „ich weiß, wie ich im Internet nützliche Gesundheitsinformationen finde“), für die die Befragten auf einer 5‑stufigen Skala ihre Zustimmung angeben konnten. Die Items wiesen eine hohe Reliabilität auf und wurden zu einem Mittelwertindex zusammengefasst (Cronbachs α = 0,926). Die Abfrage des Informationsinteresses basiert auf 3 Items einer Informationsvermeidungsskala, die ebenfalls auf einer 5er-Skala abgefragt wurden [37]. Die Items beschreiben den Wunsch nach Informationen und das Wissen über die eigene Gesundheit und gesundheitsbezogene Themen (z. B. „über meine Gesundheit will ich grundsätzlich alles erfahren“). Nach der Reliabilitätsprüfung (Cronbachs α = 0,907) wurde ein Mittelwertindex gebildet. Als Einstellung gegenüber dem Internet wurde mittels einer 5er-Skala mit den Ausprägungen „überhaupt nicht“ bis „sehr stark“ erfasst, wie vertrauenswürdig Gesundheitsinformationen aus dem Internet bewertet werden.

## Ergebnisse

Die erste Forschungsfrage fokussiert die Art und Weise der Nutzung digitaler Informations- und Unterstützungsangebote und dient dabei vor allem einer generellen Beschreibung. 72,0 % der befragten Internetnutzer*innen geben an, dass sie sich schon einmal im Internet über Gesundheitsthemen informiert haben. Innerhalb der vergangenen 30 Tage suchten diese „Gesundheits-Onliner“ an 3,37 Tagen Gesundheitsinformationen im Internet (Standardabweichung (*SD*) = 4,11). Die subjektive internetbezogene Informationskompetenz liegt im Durchschnitt im mittleren bis eher hohen Bereich (Mittelwert (*M*) = 3,44; *SD* = 0,81). Ebenso zeigt sich für das Interesse an Informationen über die eigene Gesundheit, dass es tendenziell eher hoch ausgeprägt ist (*M* = 3,58; *SD* = 1,03). Das Vertrauen in das Internet liegt im mittleren Bereich (*M* = 3,07; *SD* = 1,01). Die Bewertung fällt dabei ambivalent aus. Während 29,9 % der Befragten angeben, ein eher hohes oder hohes Vertrauen in die Gesundheitsinformationen im Internet zu haben, sind 23,9 % eher kritisch eingestellt. Unabhängig von der Recherche nach Gesundheitsinformationen im Internet, nutzen 23,8 % der Befragten auch Gesundheits-Apps.

In der Gruppe der „Gesundheits-Onliner“ dient die Onlinerecherche besonders häufig dazu, nach Krankheitssymptomen und -ursachen zu suchen (*M* = 3,18; *SD* = 0,97; Abb. 1). Dabei geben 34,9 % an, dass dies häufig bis sehr häufig das Thema ihrer Onlinerecherche ist. Bei 43,6 % ist dies zumindest gelegentlich relevant. Zudem wird im Internet zumindest gelegentlich nach Informationen über Wirkstoffe und Nebenwirkungen von Arzneimitteln und Medikamenten gesucht (*M* = 2,93; *SD* = 1,08). Sehr häufig oder häufig suchen 29,0 % der „Gesundheits-Onliner“ nach solchen Informationen. 39,4 % der Befragten geben an, dass dieses Thema gelegentlich relevant ist, bei 31,6 % ist dies selten oder nie der Fall. Tipps zu einer gesunden Lebensweise, Fitness und Wellness sind das am dritthäufigsten gesuchte Thema (*M* = 2,88; *SD* = 1,17). Es stellt bei 30,3 % ein häufiges oder sogar sehr häufiges Interesse dar und ist für weitere 34,1 % der Befragten zumindest gelegentlich bedeutsam bei der Suche nach Informationen im Internet. Die Suche nach Ärzt*innen, Therapeut*innen oder Krankenhäusern (*M* = 2,78; *SD* = 1,06) ist zumindest gelegentlich Gegenstand der Recherche im Internet. Für einen Anteil von 23,5 % der „Gesundheits-Onliner“ ist das Internet eine häufig bis sehr häufig genutzte Quelle für die Suche nach Gesundheitsexpert*innen, von 38,3 % wird es hierzu gelegentlich, von 38,1 % selten bis nie genutzt. Im Gesamtdurchschnitt eher selten befasst sich die Recherche mit Heilmethoden und Rehabilitationsmaßnahmen (*M* = 2,4; *SD* = 1,09), dem Gesundheitssystem (*M* = 2,35; *SD* = 1,08) oder Vorsorgeuntersuchungen und Früherkennungsmöglichkeiten (*M* = 2,2; *SD* = 1,09). Häufig bis sehr häufig suchen 14,9 % nach Heilmethoden und Rehabilitationsmaßnahmen, 14,3 % nach Themen, die mit dem Gesundheitssystem in Beziehung stehen, und 12,7 % der „Gesundheits-Onliner“ nach Informationen über Vorsorgeuntersuchungen.

Zu einer differenzierteren Beschreibung der Internetnutzung trägt auch die Unterscheidung der genutzten Onlineangebote bei (Abb. 2). Die durchschnittlich am häufigsten genutzten Angebote sind Gesundheitsportale (*M* = 2,68; *SD* = 1,11). 21,9 % der Befragten geben an, diese häufig oder sehr häufig aufzusuchen, 36,7 % suchen dort gelegentlich und 41,4 % selten oder nie nach Informationen. An zweiter Stelle stehen mit einer vergleichbar hohen Frequenz Online-Lexika, wie beispielsweise Wikipedia (*M* = 2,60; *SD* = 1,05). 18,8 % nutzen Lexika zumindest häufig und ein Anteil von weiteren 36,9 % gelegentlich. Internet-Apotheken (*M* = 2,56; *SD* = 1,15) werden von 20,4 % der Befragten häufig genutzt, von 34,3 % gelegentlich und von 45,3 % der Befragten nie oder nur selten. Die Webseiten von Ärzt*innen oder Krankenhäusern (*M* = 2,51; *SD* = 1,05) stellen für 16,1 % eine häufig genutzte Quelle dar, 35,2 % suchen dort gelegentlich nach Informationen und 48,7 % eher selten oder nie. Ähnliche Anteile entfallen auf die Nutzung von allgemeinen Ratgeber-Communitys oder spezifischen Gesundheits-Foren (*M* = 2,41; *SD* = 1,10) und Bewertungsportalen von Ärzt*innen (*M* = 2,36; *SD* = 1,13). 15,3 % der Befragten geben an, sich häufig oder sehr häufig an Online-Communitys zu wenden, für Bewertungsportale liegt der Anteil bei 15,5 %. Im Gegensatz dazu werden gesundheitsbezogene YouTube-Videos (*M* = 1,87; *SD* = 1,04) nur von 8,0 % und soziale Netzwerke wie Facebook oder Instagram (*M* = 1,79; *SD* = 1,08) von 9,1 % der Befragten häufig oder sehr häufig angeschaut oder genutzt. Insgesamt werden vor allem Web‑2.0‑Angebote (wie YouTube, Facebook oder Instagram) seltener genutzt als jene Angebote, die stärker auf die Bereitstellung von Gesundheitsinformationen ausgerichtet sind (z. B. Gesundheitsportale, Online-Lexika).

Die zweite Forschungsfrage zielt darauf, die personenbezogenen und motivationalen Einflussfaktoren a) der gesundheitsbezogenen Internetnutzung sowie b) der Nutzung von Gesundheits-Apps zu identifizieren. Zunächst steht im Fokus, welche Faktoren beeinflussen, ob die Bürger*innen das Internet als Quelle für Gesundheitsinformationen nutzen (Tab. 1). Für das Internet als Quelle für Gesundheitsinformationen zeigt die blockweise logistische Regressionsanalyse, dass bis auf eine derzeitige Therapie psychischer Beschwerden und dem subjektiven Gesundheitszustand alle Prädiktoren zur Erklärung der gesundheitsbezogenen Internetnutzung beitragen. Während mit steigendem Alter die relative Wahrscheinlichkeit der gesundheitsbezogenen Internetnutzung um 2 % pro Jahr sinkt, begünstigen die anderen Faktoren die Zuwendung zum Internet. Bei Frauen im Vergleich zu Männern sowie mit steigender Bildung nimmt die relative Wahrscheinlichkeit der Internetnutzung um 57 % bzw. 20 % zu. Befinden sich die Proband*innen derzeit wegen körperlicher Beschwerden in Therapie, steigt die relative Wahrscheinlichkeit der Zuwendung zum Internet um 81 %. Das Informationsinteresse (Anstieg um 30 %), die internetbezogene Informationskompetenz (Anstieg um 63 %) und das Vertrauen in das Internet (Anstieg um 50 %) beeinflussen die relative Wahrscheinlichkeit ebenfalls in erheblichem Maße. Das *R*^*2*^ nach Nagelkerke beträgt 0,216, was nach Cohen [38] einem starken Effekt entspricht. Es zeigt sich somit, dass sowohl personenbezogene als auch motivationale Faktoren wie das Interesse und subjektiv wahrgenommene Informationskompetenzen bedeutende Prädiktoren der gesundheitsbezogenen Internetnutzung sind, wobei die motivationalen Faktoren (*R*^*2*^ = 0,128) im Vergleich zu den personenbezogenen Faktoren (*R*^*2*^ = 0,088) einen höheren Einfluss auf die Zuwendung zum Internet haben.

**Table 1:** 

Variablen	Gesundheitsbezogene Internetnutzung			
	Modell 1	Modell 2		
	*B*	*B*	*Exp. (B)*	*95* *% KI*
Konstante	0,49	−2,72***	0,66	–
Alter	−0,01***	−0,02***	0,98	[0,98; 0,99]
Geschlecht (weiblich)	0,52***	0,45***	1,57	[1,32; 1,88]
Bildung	0,22***	0,19***	1,20	[1,12; 1,29]
Therapie körperlicher Beschwerden	0,72***	0,59***	1,81	[1,45; 2,25]
Therapie psychischer Beschwerden	0,05	−0,08	0,93	[0,70; 1,23]
Subjektiver Gesundheitszustand	−0,03	−0,10	0,91	[0,81; 1,03]
Informationsinteresse	–	0,26***	1,30	[1,18; 1,42]
Internetbezogene Informationskompetenz (E-Heals)	–	0,49***	1,63	[1,45; 1,84]
Vertrauen in das Internet	–	0,41***	1,50	[1,36; 1,65]
*Nagelkerkes R*^*2*^	0,088***	–	0,216***	–

Anmerkungen: *n* = 2936; blockweise logistische Regression; erster Block: personenbezogene Faktoren, zweiter Block: motivationale Faktoren

*B* Regressionskoeffizient, *Exp. (B)* Odds Ratio, *R*^*2*^ Bestimmtheitsmaß/Determinationskoeffizient, *95* *% KI* 95 % Konfidenzintervall für Exp. (B)

*** *p* ≤ 0,001, ** *p* ≤ 0,01, * *p* ≤ 0,01

Für ein vertiefendes Verständnis wird in einem weiteren Schritt für die gesundheitsbezogene Internetnutzung überprüft, inwiefern die personenbezogenen und motivationalen Faktoren bei jenen, die das Internet für gesundheitsbezogene Zwecke nutzen (die „Gesundheits-Onliner“), auch die Frequenz ihrer Internetnutzung beeinflussen (siehe Tab. 2). Die blockweise multiple Regression zeigt, dass die Nutzungshäufigkeit von allen Prädiktoren ausgenommen der Bildung signifikant beeinflusst wird. Erneut zeigt sich, dass das Alter die Frequenz tendenziell negativ beeinflusst (Regressionskoeffizient *B* = −0,05; *p* ≤ 0,001). Dies gilt ebenfalls für den subjektiven Gesundheitszustand (*B* = −0,37; *p* ≤ 0,01). Nehmen die Proband*innen ihre Gesundheit positiver wahr, nutzen sie das Internet zu gesundheitsbezogenen Zwecken seltener. Frauen nutzen das Internet häufiger (*B* = 0,45; *p* ≤ 0,01) und die Frequenz der Internetnutzung ist höher, wenn sich die Befragten derzeit wegen körperlicher (*B* = 0,81; *p* ≤ 0,001) oder psychischer Beschwerden (*B* = 0,93; *p* ≤ 0,001) in Behandlung befinden. Mit Blick auf die motivationalen Einflüsse erweisen sich ein stärkeres Informationsinteresse (*B* = 0,34; *p* ≤ 0,001), eine höhere Ausprägung der internetbezogenen Informationskompetenzen (*B* = 0,63; *p* ≤ 0,001) und ein höheres Vertrauen in das Internet (*B* = 0,38; *p* ≤ 0,001) als relevante Faktoren, die mit einer häufigeren Nutzung einhergehen. Im Vergleich der Prädiktoren zeigen die einzelnen Effektstärken, dass das Alter als Prädiktor besonders bedeutsam ist (standardisierter Regressionskoeffizient β = −0,16). Danach folgen die internetbezogene Informationskompetenz (β = 0,11) und die Therapie körperlicher Beschwerden (β = 0,10). Allerdings sind die Effektstärken als eher gering zu bewerten. Dies geht auch damit einher, dass das Gesamtmodell nur 8,8 % der Varianz in der Frequenz der gesundheitsbezogenen Internetnutzung erklären kann.

**Table 2:** 

Variablen	Frequenz der gesundheitsbezogenen Internetnutzung			
	Modell 1	Modell 2		
	*B*	*B*	ß	*95* *% KI*
Konstante	4,86***	1,14	–	[−0,27; 2,56]
Alter	−0,04***	−0,05***	−0,16	[−0,06; −0,03]
Geschlecht (weiblich)	0,53**	0,45**	0,06	[0,11; 0,79]
Bildung	0,02	−0,00	−0,00	[−0,13; 0,12]
Therapie körperlicher Beschwerden	1,01***	0,81***	0,10	[0,41; 1,21]
Therapie psychischer Beschwerden	1,08***	0,93***	0,08	[0,44; 1,42]
Subjektiver Gesundheitszustand	−0,23	−0,37**	−0,08	[−0,60; −0,13]
Informationsinteresse	–	0,34***	0,08	[0,15; 0,52]
Internetbezogene Informationskompetenz (E-Heals)	–	0,63***	0,11	[0,38; 0,87]
Vertrauen in das Internet	–	0,38***	0,09	[0,19; 0,56]
*R*^*2*^	0,053***	–	0,088***	–
*F*	20,652	–	23,779	–

Anmerkungen: *n* = 2113 (Teilsample der Personen, die das Internet zur Suche nach Gesundheitsinformationen nutzen), blockweise lineare Regression; erster Block: personenbezogene Faktoren, zweiter Block: motivationale Faktoren

*B* Regressionskoeffizient, *β* Beta/standardisierter Regressionskoeffizient, *F* F-Wert des zugrunde liegenden F‑Tests, *R*^*2*^ Bestimmtheitsmaß/Determinationskoeffizient, *95* *% KI* 95 % Konfidenzintervall

*** *p* ≤ 0,001, ** *p* ≤ 0,01, * *p* ≤ 0,01

Für die Nutzung von Gesundheits-Apps (Forschungsfrage 2b) zeigt die logistische Regression eine etwas geringere Erklärleistung (Nagelkerkes *R*^*2*^ = 0,159) der Faktoren als für die Zuwendung zum Internet, die nach Cohen [38] aber ebenfalls als stark bewertet werden kann. Neben dem fehlenden Einfluss der Therapie psychischer Beschwerden zeigt sich hier auch kein signifikanter Einfluss des Geschlechts sowie des generellen Vertrauens in das Internet (Tab. 3). Erneut ist höheres Alter mit einer geringeren relativen Wahrscheinlichkeit verbunden, eine Gesundheits-App zu nutzen, während eine höhere Bildung, die Therapie körperlicher Beschwerden ebenso wie ein besserer subjektiver Gesundheitszustand, ein höheres Informationsinteresse und eine höhere Informationskompetenz mit einer höheren Wahrscheinlichkeit der Nutzung einer Gesundheits-App einhergehen (Tab. 3). Die Frage, ob jemand eine Gesundheits-App nutzt oder nicht, wird dabei stärker durch die personenbezogenen (*R*^*2*^ = 0,124) als die motivationalen Faktoren (*R*^*2*^ = 0,035) bedingt.

**Table 3:** 

Variablen	Nutzung von Gesundheits-Apps			
	Modell 1	Modell 2		
	*B*	*B*	*Exp. (B)*	*95* *% KI*
Konstante	−1,24***	−2,83***	0,06	–
Alter	−0,04***	0,05***	0,96	[0,95; 0,96]
Geschlecht (weiblich)	−0,02	−0,07	0,93	[0,78; 1,12]
Bildung	0,13***	0,11**	1,11	[1,04; 1,19]
Therapie körperlicher Beschwerden	0,67***	0,54***	1,72	[1,38; 2,14]
Therapie psychischer Beschwerden	0,24	0,20	1,22	[0,94; 1,58]
Subjektiver Gesundheitszustand	0,31***	0,24***	1,27	[1,12; 1,44]
Informationsinteresse	–	0,11*	1,11	[1,01; 1,23]
Internetbezogene Informationskompetenz (E-Heals)	–	0,44***	1,56	[1,37; 1,77]
Vertrauen in das Internet	–	0,06	1,06	[0,96; 1,17]
*Nagelkerkes R*^*2*^	0,124***	–	0,159***	–

Anmerkungen: *n* = 2936; blockweise logistische Regression; erster Block: personenbezogene Faktoren, zweiter Block: motivationale Faktoren

*B* Regressionskoeffizient, *Exp. (B)* Odds Ratio, *R*^*2*^ Bestimmtheitsmaß/Determinationskoeffizient, *95* *% KI* 95 % Konfidenzintervall für Exp. (B)

*** *p* ≤ 0,001, ** *p* ≤ 0,01, * *p* ≤ 0,01

## Diskussion

Der Beitrag beschreibt und erklärt die gesundheitsbezogene Informationssuche deutscher Internetnutzer*innen. In einer für diese Zielgruppe nach Alter, Geschlecht, Bildung und Region stratifizierten Onlinebefragung wurde untersucht, wer nach welchen Gesundheitsinformationen im Internet sucht und welche personenbezogenen und motivationalen Einflussfaktoren die Zuwendung zu gesundheitsbezogenen Internetangeboten und Gesundheits-Apps sowie die Frequenz der Internetnutzung beeinflussen.

Die gesundheitsbezogene Internetnutzung kann als gängig und weitverbreitet gelten: Mit 72 % nutzt ein Großteil der Befragten Internetnutzer*innen in Deutschland dieses auch für die Recherche und den Austausch über Gesundheitsinformationen.^2^ Im internationalen Vergleich fällt dieser Anteil geringer aus, so liegt beispielsweise in den USA der Anteil in der Gesamtbevölkerung bei über 80 % [39]. Die hohe Verbreitung lässt sich auch auf die inhaltliche und funktionale Vielfalt der Angebote im Internet zurückführen, die z. B. bei gesundheitlichen Problemen die Möglichkeit bietet, sich rückzuversichern, eine zweite Meinung einzuholen, Arztbesuche vor- oder nachzubereiten und bei offenen Fragen schnelle Hilfestellung zu erhalten [40]. Dagegen nutzt nur etwa ein Viertel der Befragten Gesundheits-Apps. Angesichts eher spezifischer Funktionen wie des Überwachens und Dokumentierens von Verhaltensweisen mittels Apps handelt es sich hier offenbar um eine deutlich spezifischere Zielgruppe, die bisher durch das Angebot angesprochen wird.

Die Internetrecherche dient am häufigsten einem Wissenserwerb über Krankheitssymptome und -ursachen, Arzneimittel oder Medikamente sowie dem Einholen von Tipps für eine gesunde Lebensweise, Fitness und Wellness. Entsprechend breit ist das Themenspektrum, das von der Krankheitsbewältigung bis zur Gesundheitsförderung und Prävention reicht. Die hohe Bedeutung des Wissenserwerbs bzw. der Informationssuche im Vergleich zum Erfahrungsaustausch zeigen auch die genutzten Angebote. Relativ häufig werden Gesundheitsportale und Online-Lexika genutzt, während Ratgeber-Communitys und gesundheitsspezifische Online-Foren eher selten aufgesucht werden. Gesundheitsbezogene YouTube-Videos und der gesundheitsbezogene Austausch über soziale Netzwerke spielen gemessen an der Häufigkeit ihrer Nutzung nur eine untergeordnete Rolle. Die Ergebnisse stehen im Einklang mit bisherigen Untersuchungen der gesundheitsbezogenen Internetnutzung [8, 30].

Darüber hinaus wurden zunächst die Einflussfaktoren der gesundheitsbezogenen Zuwendung zum Internet und der Nutzung von Gesundheits-Apps identifiziert. Für die generelle Zuwendung zum Internet als Quelle für Gesundheitsinformationen spielen sowohl personenbezogene als auch motivationale Faktoren eine Rolle. Dabei weisen Jüngere, Frauen und höher Gebildete eine höhere Wahrscheinlichkeit der gesundheitsbezogenen Internetnutzung auf. Die Erkenntnisse bestätigen vorherige Analysen zu den Einflussfaktoren der Informationssuche [9, 22, 41]. Zudem sind es vor allem körperliche Beschwerden, die Anlass zur Suche bieten. Wer aufgrund psychischer Beschwerden in Behandlung ist, gehört hingegen nicht automatisch zu den „Gesundheits-Onlinern“. Dieser Befund passt nicht zu der dominierenden allgemeinen Annahme, dass bei psychisch Erkrankten die gesundheitsbezogene Zuwendung zum Internet per se wahrscheinlicher ist, weil es sich besonders für die Information und den Austausch über eher sensible und stigmatisierte Themen eignet [19]. Im Vergleich zu den personenbezogenen Einflüssen erweisen sich aber die motivationalen Faktoren als insgesamt noch bedeutsamer hinsichtlich dessen, ob Menschen sich dem Internet für gesundheitsbezogene Zwecke zuwenden oder nicht. Vor allem die wahrgenommenen Informationskompetenzen und das Vertrauen in das Internet sind ausschlaggebend.

Die Ergebnisse lassen Rückschlüsse sowohl auf Hintergründe von Informationsbedarfen als auch auf mögliche Barrieren zu. Bedarfe resultieren besonders aus körperlichen Beschwerden und Krankheitserfahrung. Der Einfluss der Bildung und die hohe Bedeutung der wahrgenommenen Kompetenzen verweisen auf mögliche Barrieren. Dies ist mit Blick auf die hieraus resultierende Verstärkung sozialer Ungleichheiten kritisch zu betrachten. Denn das Internet kann trotz seiner Niedrigschwelligkeit und breiten Zugangsmöglichkeiten nur dann sein Potenzial entfalten, soziale Ungleichheiten zu reduzieren, wenn die internetbezogenen Informationskompetenzen vorhanden sind. Dies unterstreicht den Bedarf an Maßnahmen zur Kompetenzförderung, wie er u. a. im Nationalen Aktionsplan Gesundheitskompetenz [42] verankert ist.

Wird für die „Gesundheits-Onliner“ die Frequenz der gesundheitsbezogenen Internetnutzung betrachtet, zeigt sich, dass die einbezogenen Faktoren einen deutlich geringeren Anteil an Varianz erklären können. Die analysierten personenbezogenen und motivationalen Faktoren scheinen somit entscheidender dafür zu sein, dass das Internet prinzipiell eine relevante Quelle für Gesundheitsinformationen darstellt, und weniger dafür, in welchem Maße es genutzt wird. Personenbezogene Faktoren erweisen sich im Vergleich zu den motivationalen Faktoren hier als bedeutsamer. Alter und Geschlecht zeigen sich auch in diesem Kontext als relevante Prädiktoren. So nutzen Jüngere ebenso wie Frauen das Internet zu Gesundheitszwecken häufiger. Die Bildung macht allerdings keinen Unterschied für die Nutzungshäufigkeit. Die Frequenz fällt zudem begleitend zur Therapie sowohl aufgrund körperlicher als auch psychischer Beschwerden höher aus, was ein anderes Muster als bei der Erklärung der Zuwendung zum Internet zeigt. Wer das Internet also zu gesundheitsbezogenen Zwecken ohnehin nutzt, tut dies bei psychischen Beschwerden häufiger. Die hohe Bedeutung eines gesundheitlichen Anlasses für die Häufigkeit der gesundheitsbezogenen Internetnutzung spiegelt sich auch in der hemmenden Wirkung eines positiven Gesundheitszustandes wider. Dies lässt den Schluss zu, dass das Internet umso häufiger als Informationsquelle und Anlaufstelle zum Austausch konsultiert wird, je höher die gesundheitliche Belastung erlebt wird, was durchaus auf Potenziale der Digitalisierung verweist. Unter den motivationalen Faktoren können ein hohes Interesse, eine höhere Ausprägung von Kompetenzen und das Vertrauen in das Internet als förderliche Faktoren einer häufigeren Nutzung angesehen werden.

Im Vergleich zur Internetnutzung zeigt sich für Gesundheits-Apps, dass deren Nutzung deutlich stärker von personenbezogenen als von motivationalen Faktoren bestimmt wird. Erneut werden vor allem Jüngere und höher Gebildete von solchen Angeboten angesprochen. Zudem deutet die hohe Bedeutung der körperlichen Beschwerden in Kombination mit einem als eher gut wahrgenommenen Gesundheitszustand darauf hin, dass solche Apps sowohl zur Unterstützung des Krankheitsmanagements und ergänzend zur ärztlichen Therapie als auch zum Erhalt und zur Steigerung von Gesundheit und Fitness genutzt werden. Auf motivationaler Ebene zeigt sich auch für die Nutzung von Gesundheits-Apps die hohe Bedeutung von Informationskompetenzen, während allgemeines Interesse eine tendenziell geringere Relevanz besitzt. Das Vertrauen in das Internet ist zudem kein signifikanter Einflussfaktor. Dies kann damit zusammenhängen, dass Gesundheits-Apps nicht zwingend als Teil des Internets angesehen werden.

Zusammenfassen lässt sich, dass sich das Internet für die Suche nach gesundheits- bzw. vor allem krankheitsbezogenen Informationen auch in Deutschland etabliert hat, während Gesundheits-Apps bislang noch von einer spezifischeren Personengruppe genutzt werden. Dabei ist die generelle gesundheitsbezogene Zuwendung zum Internet nur zum Teil von denselben personenbezogenen und motivationalen Faktoren beeinflusst wie die Intensität dieser Nutzung. Dies gilt es, nicht nur bei Analysen der Nutzung, sondern auch bei der Entwicklung und Vermittlung gesundheitsbezogener Informations- und Dialogangebote zu bedenken.

Onlineangebote werden insbesondere von weiblichen Personen, Menschen mit konkreten gesundheitlichen Beschwerden sowie hohem Interesse an Gesundheitsthemen als Informations- und Unterstützungsquelle genutzt. Hiermit verbundene Potenziale können sich aber nur dann entfalten, wenn Informationsinteresse und -kompetenzen gegeben sind – die Menschen sich also motiviert und befähigt fühlen, das Gesuchte auch zu finden – und wenn sie der Quelle vertrauen. Vor dem Hintergrund der bekannten sozialen Gradienten des Gesundheitsstatus und der Gesundheitskompetenz [42] gilt es, vor allem sozial benachteiligte Gruppen in ihrer Motivation und ihren Wirksamkeitsüberzeugungen zu unterstützen. Anderenfalls ist die Gefahr zu sehen, dass vom Zugang zu und der Nutzung von Gesundheitsinformationen im Internet nicht alle Bevölkerungsgruppen in gleichem Maße profitieren und sich gesundheitliche und soziale Ungleichheiten durch Diskrepanzen im Zugang zu Informationen sogar verstärken.

## Footnotes

1 Weitere Informationen hierzu: https://hints.cancer.gov/data/survey-instruments.aspx.

2 Für die Einordnung dieses Anteils muss beachtet werden, dass der Anteil der Internetnutzer*innen in Deutschland bei 90,3 % liegt.
