# Erratum zu: Nutzung von Gesundheitsinformationen im Internet: personenbezogene und motivationale Einflussfaktoren

**DOI:** 10.1007/s00103-021-03447-1

**Published:** 2021-10-26

**Authors:** Elena Link, Eva Baumann

**Affiliations:** Institut für Journalistik und Kommunikationsforschung, Hochschule für Musik, Theater und Medien Hannover, Expo Plaza 12, 30539 Hannover, Deutschland

**Erratum zu:**

**Bundesgesundheitsbl 2020**

10.1007/s00103-020-03144-5

Der Artikel Nutzung von Gesundheitsinformationen im Internet: personenbezogene und motivationale Einflussfaktoren von Elena Link und Eva Baumann wurde ursprünglich Online First ohne „Open Access“ auf der Internetplattform des Verlags publiziert. Nach der Veröffentlichung in Band 63, Heft 6, pp. 681–689 hatten sich die Autoren für eine „Open Access“-Veröffentlichung entschieden. Das Urheberrecht des Artikels wurde deshalb in © The Author(s) 2020 geändert. Dieser Artikel ist jetzt unter der Creative Commons Namensnennung 4.0 International Lizenz veröffentlicht, welche die Nutzung, Vervielfältigung, Bearbeitung, Verbreitung und Wiedergabe in jeglichem Medium und Format erlaubt, sofern Sie den/die ursprünglichen Autor(en) und die Quelle ordnungsgemäß nennen, einen Link zur Creative Commons Lizenz beifügen und angeben, ob Änderungen vorgenommen wurden.

Die in diesem Artikel enthaltenen Bilder und sonstiges Drittmaterial unterliegen ebenfalls der genannten Creative Commons Lizenz, sofern sich aus der Abbildungslegende nichts anderes ergibt. Sofern das betreffende Material nicht unter der genannten Creative Commons Lizenz steht und die betreffende Handlung nicht nach gesetzlichen Vorschriften erlaubt ist, ist für die oben aufgeführten Weiterverwendungen des Materials die Einwilligung des jeweiligen Rechteinhabers einzuholen.

Weitere Details zur Lizenz entnehmen Sie bitte der Lizenzinformation auf http://creativecommons.org/licenses/by/4.0/deed.de.

